# Automated Text Message–Based Program to Improve Uncontrolled Blood Pressure in Primary Care Patients: A Randomized Clinical Trial

**DOI:** 10.1007/s11606-024-09225-4

**Published:** 2024-12-04

**Authors:** Eric Bressman, Klea Profka, Laurie Norton, Kayla Clark, Katy Mahraj, Zakiya Walker, Leslie Reid-Bey, Anthony Girard, Charles Rareshide, Lin Xu, Jingsan Zhu, Mary Putt, Kevin G. Volpp, Anna U. Morgan

**Affiliations:** 1 Department of Medicine, Perelman School of Medicine, University of Pennsylvania, Philadelphia, PA USA; 2 Leonard Davis Institute of Health Economics, University of Pennsylvania, Philadelphia, PA USA; 3 Center for Health Incentives and Behavioral Economics, University of Pennsylvania Health System, Philadelphia, PA USA; 4 Department of Medical Ethics and Health Policy, University of Pennsylvania, Philadelphia, PA USA; 5 Center for Health Care Transformation and Innovation, University of Pennsylvania Health System, Philadelphia, PA USA; 6 Department of Biostatistics, Epidemiology, and Informatics, Perelman School of Medicine, University of Pennsylvania, Philadelphia, PA USA

## Abstract

**Background:**

Suboptimal control of BP is common, although safe and effective treatments are widely available. Conventional management relies on office visits, but this can be an inefficient path to medication optimization.

**Objective:**

To assess the effectiveness of an intensive, 6-month remote BP management program among patients with uncontrolled hypertension.

**Design:**

A two-arm randomized clinical trial which ran from January to July 2023 at two primary care practices with an in-clinic BP measurement at the end of the intervention.

**Participants:**

Established adult patients (ages 21–80) of study practices with uncontrolled hypertension (two measurements > 140/90 in the prior 12 months) and an active prescription for at least one anti-hypertensive agent.

**Intervention:**

Participants received automated text messages prompting them to check their BP weekly for 6 months. An RN and APP monitored BP data entered by the participant. The automated platform escalated any out-of-normal range readings or needs to the program staff.

**Main Measures:**

The primary outcome was change in SBP from baseline to the end-of-study measurement. Enrollment and engagement measures were collected for the intervention arm.

**Key Results:**

Of the 300 participants, the mean (SD) age was 63 (± 12.2) years; 133 (44.3%) were male and 167 (55.7%) were female; 154 (51.5%) self-identified as Black and 120 (40.1%) White; and 119 (39.7%) were insured by Medicare and 41 (13.7%) by Medicaid. The change in SBP at 6 months among those who completed the end-of-study measurement was − 14.66 mmHg (95% CI − 19.95, − 9.36) in the intervention arm and − 10.87 mmHg (95% CI − 18.04, − 3.69) in the control arm (*p* = 0.39). Within the intervention arm, 97 participants (64.7%) completed all enrollment steps, and these participants submitted BPs 72.8% of the weeks. Participants in the intervention arm had a greater number of medication changes (0.81 vs 0.57 in the control arm, *p* = 0.01) over the study period.

**Conclusions:**

In this randomized clinical trial of a 6-month automated text messaging program, there was no significant difference in the change in SBP among participants in each arm.

**Trial Registration:**

ClinicalTrials.gov Identifier: NCT05571410.

**Supplementary Information:**

The online version contains supplementary material available at 10.1007/s11606-024-09225-4.

## INTRODUCTION

Hypertension is the world’s most common risk factor for atherosclerotic cardiovascular disease and is a leading cause of disability adjusted life years.^1^ Hypertension affects 29% of US adults and 63% of older adults.^2^ Safe and effective treatments are widely available; however, use of these treatments and optimal titration is limited by a reliance on conventional, face-to-face appointments.^3^

This is limited for a number of reasons: (1) Office visits are often arranged to address more urgent issues that garner patient and clinician attention; (2) office measurements provide a restricted window into true BP control (both because of infrequent data points and discrepancies between office and home measurements);^4,5^ (3) the episodic nature of this approach slows the rate of medication titration;^6^ and (4) office visits can hinder patient engagement. Remote patient monitoring, supported by bidirectional text messaging, may help overcome these limitations. Text messaging can improve engagement, with patients more likely to measure and submit BPs than via office follow-up or other modes of outreach.^7,8^ This can support a more efficient pathway to control, with increased data points and opportunities to titrate medications.

Frequent communication with patients regarding BP can create additional work for clinicians. An automated texting program could solve some of the workload challenges posed by a remote monitoring program by triaging messages and applying pre-specified rules to incoming BP data. Staff can be notified only when there are abnormalities requiring direct clinician involvement.

We developed a remote BP management program, called BP Pal, designed to provide automated patient-facing feedback with clinician involvement only as necessary. The program included automated, bidirectional text messaging program to facilitate patient engagement; BP data collection and processing; and a human backend for direct clinical management, including medication adjustments. We hypothesized that this approach would lead to improvements in BP among patients with uncontrolled hypertension. We tested the feasibility and effectiveness of this program in a randomized clinical trial across two primary care practices in a single academic health system.

## METHODS

### Overview

We conducted a two-arm randomized clinical trial between January and July 2023. Participants were assigned to either usual care or usual care plus a 6-month remote monitoring program via automated text messaging. The texting program was built and managed by Way to Health (W2H), a Penn-based software platform created with funding from the NIH to provide automated technology infrastructure in support of clinical care and research (see Appendix for the complete protocol). This study was reviewed and approved by the University of Pennsylvania Institutional Review Board. We followed the Consolidated Standards of Reporting Trials Extension (CONSORT Extension) reporting guideline (with pragmatic extension).

### Participants and Study Sites

The patient population is comprised of adults (ages 21–80) actively followed by one of two Penn Medicine primary care practices. Inclusion criteria were (1) uncontrolled hypertension (two office-based BPs > 140/90 mmHg in the last 12 months, including most recent) and (2) an active prescription for at least one anti-hypertensive agent. Patients were excluded if they were (1) pregnant or breastfeeding, (2) had a significant disability or markedly shortened life expectancy (metastatic cancer, on hospice, end-stage renal disease, dementia) or (3) were unable to text in English.

### Enrollment Procedures and Randomization

Participants were identified for inclusion through the electronic health record. A total of 587 eligible participants were initially identified as eligible and transmitted to primary care clinicians for potential opt-out (prior to randomization). Overall, 24 of those eligible were opted out during this review process. The final sample was randomly selected from the remaining list to meet our target size and uploaded into the W2H platform for randomization and enrollment.

The trial received a waiver of consent given that (1) both arms received usual care, (2) text outreach involved no more than minimal risk to participants, (3) participants were free to opt out at any time, and (4) requiring informed consent would significantly limit the external validity of the study.

**Figure 1:**
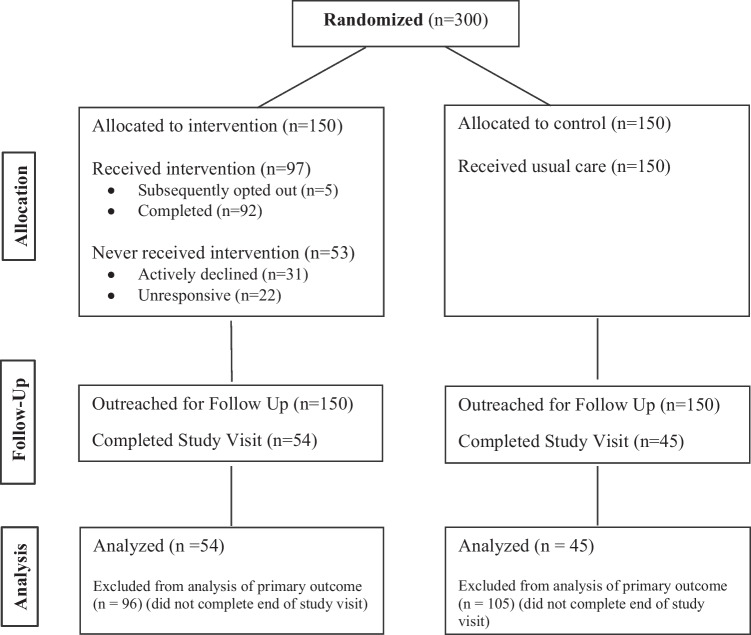
Consort diagram.

Participants were randomized 1:1 to the intervention or control arm, and randomization was balanced by practice (Fig 1). Investigators and data analysts were blinded to assignment, but clinical staff were not.

### Control Arm

Participants in the control arm did not receive any component of the program but continued to receive usual care by their primary care clinicians.

### Intervention Arm

Participants in the intervention arm were enrolled in BP Pal in addition to usual care. Upon enrollment, participants received an introductory text message describing the program and advising them on how to opt out or contact the study team. Participants who did not opt out were prompted to verify their home address and select either a regular or large-sized cuff. BP monitors were mailed to participants along with instructions on how to set up the monitor and accurately measure BP. Study coordinators called participants who did not send BPs to troubleshoot.

Participants received an automated text reminder to submit a BP every Tuesday. After 3 weeks in the program, participants were offered to reschedule the time of the reminder. Participants who submitted out-of-range BP values (see Appendix for clinical protocol) received additional messaging tailored to the BP category (high/low, critical/non-critical) and were asked to send a repeat measurement. If the second BP was also abnormal, an “escalation” alert was sent to the study RN via the Epic in-basket (with urgency dependent on the BP category), which was monitored during regular business hours.

A custom-built interface displayed longitudinal BP data and flagged abnormal trends. This was reviewed weekly by the study’s core clinical dyad (RN/APP), with support from the study’s medical director (physician) as needed.

The study RN contacted participants via phone in response to (1) “escalations,” as described above (typically single, critical out-of-range values which require more immediate follow-up; see the Appendix for further detail) or (2) abnormal trends (typically multiple, non-critical out-of-range values over time). Symptoms were assessed and medications were adjusted at the discretion of the study’s clinical dyad.

Participants could opt out at any time. Participants who did not respond to three consecutive weekly reminders received an additional message asking whether they needed help or no longer wanted to participate. If participants did not respond to this, they continued to receive the messages. Participants received a 1-item satisfaction survey at month 6.

### End of Study Visit

At month 5, all participants randomized received a text with instructions about an end-of-study, in-office BP check with a link to schedule. Participants were offered $95 to complete the visit. Participants who had questions were instructed to text HELP. Uninterested participants could opt out by texting NO or BYE. Study coordinators contacted participants who did not schedule an appointment. At this visit, participants had 3 BP measurements taken 5 min apart, and the final value was the average of the last two.

### Measures and Outcomes

The primary outcome was the change in SBP from baseline (the most recent measure prior to study start) to the 6-month study visit. Pre-specified secondary outcomes included the change in systolic and diastolic BP from baseline to approximately 3 months (between months 2 and 4) and 6 months (between months 5 and 7) post randomization, as captured from non-study ambulatory visits in the EHR; and the proportion of patients completing the study visit who achieved BP control (< 140/90). Additional exploratory outcomes include the number of medication changes (extracted from the EHR and defined as any change in dose, addition, or discontinuation of an anti-hypertensive medication) and changes in BP between the first and last home SBP readings for those in the intervention arm.

For the intervention arm, additional engagement measures were obtained from the W2H platform. These included enrollment and opt-out rates, the percentage of participants enrolled in the intervention submitting at least one BP measurement, and the average number of total weeks in the intervention in which participants submitted a BP reading. Participant satisfaction with the program was measured via the Net Promoter Score.^9^ This 1-item survey elicits a score ranging from 0 (unlikely) to 10 (extremely likely) in response to the question: “On a scale of 1 to 10, how likely are you to recommend BP Pal to a friend or family member who needs help with blood pressure monitoring?” The overall score is reported as an integer value from − 100 to + 100 (see Appendix for further details on scoring). Finally, for patients enrolled in the intervention, we report the change in SBP from the following: (a) baseline to first home BP, (b) baseline to last home BP, and (c) first to last home BP.

### Analysis

The target sample size was 300 participants based on (1) a projected 33% attrition rate (between participants opting out and/or not completing the end-of-study visit), drawn from prior experience, and (2) 80% power and a two-sided significance level of 0.05 to detect a mean systolic pressure difference of 7 mmHg between the study arms. Analyses were conducted using an intention-to-treat approach. For the main analysis, we used the study measures collected, as specified. As an additional exploratory approach, we conducted multiple imputation using the mice package with 20 imputations for BP data that were missing (no end-of-study visit or 6-month EHR measure available) and pooled results across imputed datasets using Rubin’s Rule.^10^ Continuous outcomes, including the primary outcome, were compared between intervention and control arms using a two-sample *t* test. Binary outcomes were compared using Pearson’s chi-squared test.

All statistical analyses were performed using R 4.2.2.

## RESULTS

### Study Population

A total of 300 participants were enrolled and randomly assigned to the intervention (*n* = 150) or control (*n* = 150) arm, among which the mean (SD) age was 63 (12.2) years; 133 (44.3%) were male and 167 (55.7%) were female; 154 (51.5%) Black, and 120 (40.1%) White; 119 (39.7%) were insured by Medicare and 41 (13.7%) by Medicaid (Table 1). The mean starting SBP was 152.4 (12.6), and the mean baseline number of anti-hypertensive medications used by participants was 2.1 (1.2).

**Table 1:** Patient Characteristics

Characteristic	Intervention (*N* = 150)	Control (*N* = 150)	Overall (*N* = 300)
Age, median [IQR]	64 [55, 71]	61 [51, 71]	63 [53,71]
Sex, no. (%)			
Male	60 (40.0)	73 (48.7)	133 (44.3)
Female	90 (60.0)	77 (51.3)	167 (55.7)
Race, no. (%)			
Asian	6 (4.0)	5 (3.4)	11 (3.7)
Black	76 (50.7)	78 (52.3)	154 (51.5)
Other^a^	7 (4.7)	7 (4.7)	14 (4.7)
White	61 (40.7)	59 (39.6)	120 (40.1)
Ethnicity, no. (%)			
Hispanic or Latino	6 (4.0)	4 (2.7)	10 (3.3)
Non-Hispanic, Non-Latino	144 (96.0)	146 (97.3)	290 (96.7)
Insurance type, no. (%)			
Commercial	65 (43.3)	73 (48.7)	138 (46.0)
Medicaid	22 (14.7)	19 (12.7)	41 (13.7)
Medicare	63 (42.0)	56 (37.3)	119 (39.7)
Self-pay	0 (0.0)	2 (1.3)	2 (0.7)
PCP department, no. (%)			
Westtown	75 (50.0)	77 (51.3)	152 (50.7)
University City	75 (50.0)	73 (48.7)	148 (49.3)
Charlson Comorbidity Index, mean (SD)	2.27 (2.24)	2.26 (2.39)	2.27 (2.31)
Hypertensive agents, no. (%)	2.14 (1.18)	2.13 (1.17)	2.13 (1.17)
Baseline SBP, mean (SD)	152.4 (11.4)	152.3 (13.7)	152.4 (12.6)
Baseline DBP, mean (SD)	84.6 (9.9)	86.4 (12.4)	85.5 (11.2)

^a^Includes American Indian and Alaskan Native, and unknown/answered “other”

### Primary Outcome

All participants randomized (*n* = 300) were invited for an end-of-study BP check, of whom 99 (33.0%) completed visits (54 in the intervention and 45 in the control arm). Among these participants, the change in SBP at 6 months was − 14.66 mmHg (95% CI − 19.95, − 9.36) in the intervention arm and − 10.87 mmHg (95% CI − 18.04, − 3.69) in the control arm; this difference was not statistically significant (*p* = 0.39) (Table 2). Of note, within this group, the starting SBP was greater for those in the intervention arm (153.74) than those in the control arm (148.80).

**Table 2:** Primary and Secondary Outcomes

		Intervention				Control				*p* values
		*N*	Pre	Post	Change (95% CI)	*N*	Pre	Post	Change (95% CI)	
Primary	Change in SBP at 6 months (study visit)^a^	54	153.74	139.08	− 14.66 [− 19.95, − 9.36]	45	148.80	137.93	− 10.87 [− 18.04, − 3.69]	0.39
Secondary	Change in SBP at 3 months (EHR)^b^	80	151.34	139.05	− 12.28 [− 16.03, − 8.54]	63	151.46	142.62	− 8.84 [− 13.98, − 3.69]	0.28
	Change in DBP at 3 months (EHR)^b^	80	83.92	78.76	− 5.16 [− 7.67, − 2.65]	63	84.54	81.29	− 3.25 [− 6.27, − 0.22]	0.33
	Change in SBP at 6 months (study visit + EHR)^c^	102	152.57	139.19	− 13.37 [− 16.89, − 9.86]	93	151.73	138.39	− 13.34 [− 17.95, − 8.73]	0.99
	Change in DBP at 6 months (study visit + EHR)^c^	102	83.80	81.26	− 2.55 [− 4.52, − 0.58]	93	86.41	81.27	− 5.14 [− 7.90, − 2.38]	0.13
	Change in SBP at 6 months (study visit + EHR + imputation)^d^	150	152.39	139.44	− 12.94 [− 16.32, − 9.57]	150	152.32	137.85	− 14.47 [− 18.52, − 10.42]	0.57
	BP controlled at end of study visit, no. (%)	54	24 (44.4%)			45	23 (51.1%)			0.646

^a^Includes only those patients who completed the in-person, end-of-study visit

^b^EHR-documented BP data collected from office visits at or around 3 months from the start of the study period

^c^For patients who completed the end-of-study visit, this BP data was used; for those who did not, but did have an office-based measurement in the EHR at or around 6 months, this data was used

^d^Imputed remaining missing values for individuals who did not have either an end-of-study visit or an office-based measurement in the EHR during the specified time period

When including non-study office visit measurements (*n* = 195), the change in SBP at 6 months was − 13.37 (95% CI − 16.89, − 9.86) and − 13.34 mmHg (95% CI − 17.95, − 8.73) in the intervention and control arms, respectively (*p* = 0.99). When using multiple imputation for the remaining missing values (*n* = 300), the change in SBP at 6 months was − 12.94 (95% CI − 16.32, − 9.57) and − 14.47 mmHg (95% CI − 18.52, − 10.42) in the intervention and control arms, respectively (*p* = 0.57).

### Secondary Outcomes

At 3 months from the start of the study, the change in SBP (*n* = 143) was − 12.28 mmHg (95% CI − 16.03, − 8.54) and − 8.84 mmHg (95% CI − 13.98, − 3.69) in the intervention and control arms, respectively (*p* = 0.28) (Table 2). The change in DBP at 3 months was − 5.16 (95% CI − 7.67, − 2.65) and − 3.25 mmHg (95% CI − 6.27, − 0.22) in the intervention and control arms, respectively (*p* = 0.33). The change in DBP at 6 months (*n* = 195) was − 2.55 (95% CI − 4.52, − 0.58) and − 5.14 mmHg (95% CI − 7.90, − 2.38) in the intervention and control arms, respectively (*p* = 0.13).

Among participants who completed the end-of-study visit, the proportion with controlled BP was not significantly different between the control (51.1%) and intervention arms (44.4%) (*p* = 0.65).

### Exploratory Secondary Outcomes

The mean number of medication changes was 0.81 (0.87) in the intervention arm and 0.57 (0.85) in the control arm (*p* = 0.01), though the net change in absolute number of medications was similar (Appendix). Among participants in the intervention arm who sent in at least two BPs (*n* = 83), the mean baseline (office based) SBP was 152.87 (12.70), the mean first home SBP was 136.88 (17.65), and the mean last home SBP was 131.0 (12.41) (Appendix).

### Engagement and Satisfaction Measures

Within the intervention group, 97 participants (64.7%) completed enrollment in the program (Table 3). Of those who did not enroll, 31 (20.1%) declined to participate in the program and 22 (14.7%) were unreachable by phone and text message. An additional 5 opted out prior to the end of the intervention. A total of 86 participants (57.3% of those in the intervention arm) sent in at least 1 BP, and they submitted BPs 72.8% of the weeks in the program; in other words, 41.7% of potential person-weeks in the program had BP data submitted.

**Table 3:** Patient Engagement (Intervention Arm Only)

Observations	150
Enrolled, no. (%)	97 (64.7)
Declined enrollment, no. (%)	31 (20.1)
No response, no. (%)	22 (14.7)
Engaged (sent at least 1 BP), no. (%)	86 (57.3)
BP submission rate, mean (SD)^a^	72.8 (26.6)
NPS response, no. (%)^b^	45 (48.9)
NPS score	+ 76

^a^Among patients enrolled in the intervention (*n* = 97), the percent of weeks in which they submitted a BP

^b^Of those who completed the program and received the NPS survey question (*n* = 92)

Of those that received the NPS survey question at the end of the program (*n* = 92), 45 (48.9%) responded, for an NPS score + 76. All randomized participants (*n* = 300) were invited to complete the in-office, end-of-study BP check; 132 (44.0%) scheduled and 99 (33.0%) completed visits.

## DISCUSSION

In this randomized trial of a 6-month remote BP management program supported by two-way automated texting and a centralized care team, the patients in the intervention arm did not achieve significantly better reduction in SBP than those receiving usual care.

Several features of our program differ from those previously studied. One is the use of bi-directional, automated text messaging. Text messaging is low cost and widely used^10^ and has been associated with higher rates of engagement than phone calls and patient portals.^7,11,12^ While other programs have used remote monitoring to promote self-management of hypertension,^13–16^ our program leveraged a centralized model of care, supported by automation, to enable rapid medication titration. Inbound BP data and longitudinal trends were triaged automatically based on pre-set clinical protocols, which meant the staff’s effort was dedicated to monitoring actionable information: (1) clinically urgent values (hypotension, severe hypertension) and (2) reviewing longitudinal BP data that was labeled to highlight abnormal trends. Active management of BP medications was higher in the intervention arm, although on balance there was no significant difference in the total number of medications at the end of the study period.

Despite these features, the intervention did not perform significantly better than usual care. Both groups saw large drops in BP, and it is possible that the novelty of the model of care tested here, while conceptually appealing, does not offer significant benefit over usual care, which has evolved over time to offer tailored approaches to patients with poorly controlled BP. A recent meta-analysis of application-based remote BP management programs found a mean improvement in SBP of − 4.0 mmHg compared to control, although there was wide variability across studies.^17^ This is similar in magnitude to our findings (− 14.7 in the intervention vs − 10.9 in the control, or a difference of − 3.8), though the difference was not statistically significant. An important limiting factor was the attrition in our primary analytic sample (those who came in for the end-of-study visit). While we had anticipated a one-third attrition rate, we saw twice that, leaving the study relatively underpowered.

The pragmatic design of our study was both a strength and limitation. Enrollment was a streamlined process, which relied on a waiver of informed consent: participants were identified as eligible in the EHR, uploaded to the program, and randomized. Those assigned to the intervention arm received an introductory text message and, after verifying their identity and address, were sent a BP monitor to begin the program. This design reflects how the program might operate at scale as a population health strategy. Prior studies have required that patients complete informed consent,^13,18,19^ which increases engagement among study subjects but limits generalizability to real-world practice.^20^ In our intervention only 57.3% of participants sent BPs; among this group, BPs were submitted 72.8% of potential weeks. Across the entire intervention arm, data was submitted for 41.7% of potential person-weeks, limiting the program’s potential impact.

Another phenomenon to highlight is regression to the mean, which is commonly observed with longitudinal BP monitoring.^21,22^ We attempted to mitigate this by requiring multiple high measurements in the year prior to enrollment. However, among participants in the intervention arm who submitted home BPs, there was a 16.5 mmHg drop in SBP between their baseline (office-based) and first home measurement. While there have been observed differences in office and home-based measurements,^23^ this is a larger difference than is typically seen. Participants in the intervention arm who submitted BPs had an additional 6 mmHg decrease over the course of the study, but most of the gains occurred before the program started. Future studies of remote monitoring programs should consider a run-in phase to establish a true home baseline for comparison.

Finally, while the intervention may not have outperformed usual care with respect to BP control, it does present an alternative model for BP management. The distinctive elements—including automation, text messaging, and a centralized BP management team—could make this an operationally efficient approach for practices looking to carve out hypertension care from the busy agenda of most primary care visits.

## LIMITATIONS

Limitations of this study, in addition to those highlighted above, include that it was conducted within a single academic health system, though among a diverse patient population. The model was reliant on a single RN-APP dyad, which may hinder generalizability. Primary outcome collection was limited to those who came for the end-of-study visit; these participants may have differed from those who did not come in. We were limited in our ability to assess the efficiency of this approach relative to usual care. We similarly did not have access to data on intermediate measures, such as medication adherence, which may have revealed a more proximal effect of the intervention.

## CONCLUSION

In this randomized controlled trial of a 6-month remote management program supported by automated text messaging, we found no significant difference in BP change from usual care. Patient engagement was only moderate, limiting potential effectiveness. Satisfaction with the program among engaged participants was high, and automation supported the work of the centralized care team. Future study should explore the efficiency of this model relative to usual care.

## Supplementary Information

Below is the link to the electronic supplementary material.

Supplementary file1 (DOCX 140 KB)

Supplementary file2 (DOC 373 KB)

Supplementary file3 (DOCX 41 KB)
