# Supplementary material for: Automated Text Message–Based Program to Improve Uncontrolled Blood Pressure in Primary Care Patients: A Randomized Clinical Trial
Source: J Gen Intern Med. 2024 Dec 4;40(6):1248–54. doi: 10.1007/s11606-024-09225-4 (PMC12045849; doi:10.1007/s11606-024-09225-4)
Supplement: Supplementary file 1 — Supplementary file1 (DOCX 140 KB) [file 11606_2024_9225_MOESM1_ESM.docx]

**Supplementary Methods Appendix**

**Supplement to:** Automated Text Message-Based Program to Improve Uncontrolled Blood Pressure in Primary Care Patients: A Randomized Clinical Trial

This appendix has been provided by the authors to give readers additional information about the work.

**Table of Contents**

1. Clinical Protocol
2. Messaging Script
3. Table 1. Medication Changes Among the Intervention and Control Arms
4. Table 2. SBP among those completing at least 2 home measurements (intervention arm only)

**Methods:**

Clinical Protocol:


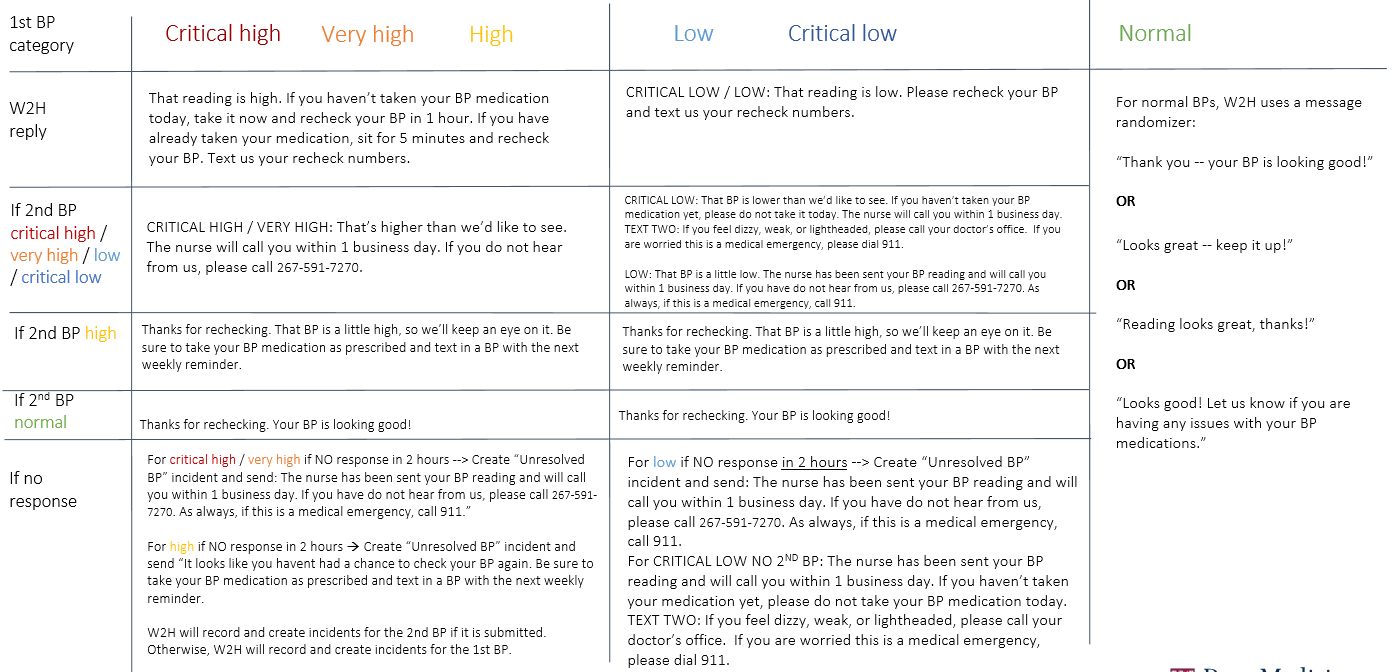


Script of Messages:

1. Enrollment messages:
   1. Initial Outreach Message: Hi [Participant Name], this is a message from the team of [PCP] at Penn Medicine. We are writing to let you know about a text message program that we think would be helpful to your health. Please text YES to confirm you are [Participant Name] and learn more about the program. If you do not want texts about the program, text NO. Texting is not 100% secure. Msg & data rates may apply.
   2. More information and Address Confirmation: Welcome to BP Pal! A spot is reserved for you with this free service from [PCP] to improve blood pressure control. We will send you a free BP cuff and text you once a week to check your BP for the next 6 months. We will monitor your BP from home and may adjust your medication based on your numbers. Text HELP with questions or BYE to unenroll at any time. Please confirm your mailing address for the BP cuff: [Address] Text YES if correct or NO if wrong.
   3. BP Monitor Sizing: Thanks for confirming your address. BP cuffs come in two sizes. Regular fits arms 9-17 inches around and long fits arms 17-19 inches around. Reply 1 if you need a regular cuff, 2 if you need a long cuff, or HELP if you have questions. 1) regular cuff 2) long cuff
   4. Final Enrollment Message with More Information: We're preparing your BP monitor! Please use the monitor that we are shipping you for this program, even if you already have one at home. Be sure to save this phone number in your contacts as BP Pal. Our business hours are Monday-Friday 9am-5pm. Visit https://w2h.us/bppal to learn more. Thanks for joining BP Pal!
2. Regular Check-In Message:
   1. Initial Check-In: Hi [Participant Name], we hope you are having a good day. This is your reminder to check and send us your blood pressure reading today (ex. 120/80).
   2. Reminder Message: Good morning! We haven’t received your BP this week. Please take a moment to check your BP and text us back.
3. Scheduling End of Study Visit Message:
   1. Control Arm: Hi [Participant Name], controlling your blood pressure is an important part of heart health. Click here: https://w2h.us/bppalscheduler to schedule a one-time BP check to help us study and improve how we manage blood pressure. For your time and effort, you’ll be compensated $95. Questions? Text HELP and we’ll call you.
   2. Intervention Arm: Congratulations, [Participant Name], you have reached your last month with BP Pal! It’s time to schedule an appointment at your primary care provider’s office using this link: https://w2h.us/bppalscheduler. You will be compensated $95 for your time. Questions? Text HELP and we’ll call you.
4. Closing Message:
   1. Congratulations, [Participant Name]! You have completed BP Pal! We will send a message to your primary care doctor to let them know the good news. Keep taking your medications as prescribed. We are proud to have worked with you to improve your heart health. You can continue to use your BP monitor to check your BP from home. We will NOT be monitoring your BP by text. If you have questions about your BP in future, please contact your primary care doctor’s office. Thank you for being part of the program!
5. Net Promoter Score Question:
   1. On a scale of 0 (unlikely) to 10 (extremely likely), how likely are you to recommend BP Pal to a friend or family member who needs help with blood pressure monitoring?

Calculation of Net Promoter Score (NPS)

1. NPS = % promoters - % detractors
   1. “Promoters” are those who give a score of 9-10
   2. “Detractors” are those who give a score of 0-6
   3. “Passives” are those who give a score of 7-8
2. The score is reported as an integer value from -100 to +100

**Table 1.** **Medication Changes Among the Intervention and Control Arms**

|  | Intervention | Control | P-Value |
| --- | --- | --- | --- |
| No. of medication changes, mean (SD)^a^ | 0.81 (0.87) | 0.57 (0.85) | 0.01 |
| No. of medications prior, mean (SD) | 2.14 (1.18) | 2.13 (1.17) | 0.92 |
| No. of medications post, mean (SD) | 2.25 (1.23) | 2.21 (1.17) | 0.81 |

^a^ Medication changes include starting a new medication, dropping a prior medication, or changing the dose of prior medication.

**Table 2. SBP among those completing at least 2 home measurements (intervention arm only)**

| Observations | 83 |
| --- | --- |
| Baseline, mean (SD)^a^ | 152.87 (12.70) |
| First home measurement, mean (SD) | 136.88 (17.65) |
| Final home measurement, mean (SD) | 131.04 (12.41) |

^a^ Office-based measurement
