# Supplementary material for: Automated Text Message–Based Program to Improve Uncontrolled Blood Pressure in Primary Care Patients: A Randomized Clinical Trial
Source: J Gen Intern Med. 2024 Dec 4;40(6):1248–54. doi: 10.1007/s11606-024-09225-4 (PMC12045849; doi:10.1007/s11606-024-09225-4)
Supplement: Supplementary file 2 — Supplementary file2 (DOC 373 KB) [file 11606_2024_9225_MOESM2_ESM.doc]

**Social and Behavioral Sciences Human Research Protocol Template 9/01/2022**

**PRINCIPAL INVESTIGATOR:** Anna Morgan M.D.

**PROTOCOL TITLE:** Program Design Hypertension Chat Bot Pilot Study with the Primary Care Service Line

**Abstract:**

A 2-arm randomized pilot trial aimed at evaluating the effectiveness of a 6-month remote monitoring program for the management of hypertension (HTN). 300 eligible patients will be randomized in a 1:1 ratio into two recruitment arms: A) Control (usual care), or B) Remote monitoring via automated messaging program. The target population are patients at the Penn Family Medicine University City, Penn Presby and Westtown primary care practices with baseline poor blood pressure control as indicated by two blood pressures greater than 140/90 within the past twelve month including the most recent measurement and on at least one anti-hypertensive agent.

**OBJECTIVES:**

To examine the effectiveness of the chatbot supported remote monitoring program (intervention) versus usual care (control) on blood pressure control among primary care patients with poorly controlled hypertension.

Hypothesis 1a: The intervention arm will see a greater reduction in systolic blood pressure than the control arm.

Hypothesis 1b: A greater proportion of patients in the intervention arm will have well controlled blood pressure than those in the control arm.

**BACKGROUND:**

Hypertension is the world’s most common risk factor for atherosclerotic cardiovascular disease and is a leading cause of disability-adjusted life-years.^1^ In the US, hypertension affects 29% of all adults and 63% of older adults.^2^ Unlike many other diseases, safe, effective, inexpensive, and evidence-based treatments are widely available for hypertension. The implementation of these treatments is often limited by a reliance on conventional face-to-face appointments, which is ineffective as the sole basis of a population health strategy.^3^

One promising approach to augment face to face visits has centered on using remote monitoring supported by bidirectional text messaging. A text message supported approach has shown improved engagement, with patients more likely to measure and submit blood pressures, than other modes of outreach.^4,5^ At the same time, a relatively frictionless communication channel presents a conundrum for providers: frequent communication regarding blood pressure monitoring can result in better outcomes but also create more work.

A chatbot with natural language processing (NLP) capability could solve the workload challenges of bidirectional texting while facilitating engagement. We have developed a chatbot and piloted it in the Penn employee hypertension management program, demonstrating both acceptability and feasibility**.** The goal of this study is to test the program’s effectiveness in a pragmatic randomized controlled trial among primary care patients with hypertension, including in a West/Southwest Philadelphia lower SES population.

**CHARACTERISTICS OF THE STUDY POPULATION:**

***1. Target Population and Accrual:***

Patients of the primary care practices listed below who have baseline poor blood pressure control will be reached out to for this study. The study will be conducted among patients seen by Penn Primary Care Providers (PCP).

Participants in this study will be identified via electronic medical records review by our study staff. The study staff will work with UPHS to create a filter for the electronic medical record system that will generate a list of patients who may meet the study criteria. A study coordinator will review the list of patients to determine eligibility. If patients meet the study requirements, they will be included on a list that will be sent to their primary care provider for review. PCPs will have the opportunity to opt any patients out of study enrollment. Patients who are not opted out by their PCP will be randomized and recruited according to study arm.

***2. Key Inclusion Criteria:***

1) Two blood pressures > 140/90 within the past 12 months, including the most recent measurement

AND

2) On at least one anti-hypertensive agent

AND

3) On HTN registry

AND

4)On PCSL registry

5) Are active patients at the Penn Family Medicine University City, Penn Presby and Westtown pratices

***3. Key Exclusion Criteria:***

Any patients meeting the following criteria are excluded from the study:

- On a PCSK9 inhibitor medication
- Pregnant or currently breastfeeding
- Have a significant disability or markedly shortened life expectancy (metastatic cancer, on hospice, ESRD, dementia, end stage renal failure, congestive heart failure)
- Not fluent in English

*.*

***4. Subject Recruitment and Screening:***

Patients are identified through an Epic Clarity report which is run as needed, until enrollment is completed. This patient list is based on the eligibility criteria. This report will be sent to the research team.

The Research team will send PCPs a list of their patients that are being considered for enrollment through Epic in-basket messages. They will be given the opportunity to identify patients who should not be enrolled.

The patients that are not opted-out by their PCP will then be randomized to the intervention or control arm.

The message sent to the PCP can be found in the attached Patient and Provider Materials document.

## *5. Early Withdrawal of Subjects:*

The participants enrolled into the intervention will be free to opt-out at any time (they can do so by texting in “bye” or “stop”). Instructions on how to do so will be clearly stated in the introductory message. The PI may withdrawal a subject if it deemed the participant is no longer an appropriate fit for the study.

***6. Vulnerable Populations:***

No vulnerable populations are specifically included or excluded in the research study. We will include all eligible patients.

***7. Populations vulnerable to undue influence or coercion:***

We are not specifically targeting any vulnerable populations.

**STUDY DESIGN:**

Pragmatic, non-blinded, randomized controlled trial.

Randomization and enrollment:

- Randomization will be at the patient level (stratified/balanced randomization by PCP)
- Enrollment will take place over short time period based off of lists of eligible patients pulled from the EMR
- Lists of eligible patients sent to PCPs with opportunity to opt patients out. Patients will be contacted via text followed by phone call to introduce them to the program.

We expect recruitment to take approximately 1 month to reach a target sample size of 300. The program itself will be 6 months from the date of randomization. Total study duration should be approximately 7 months. Data analyses will take an additional 3 months.

The control group will receive usual care as determined by PCP (control arm will not receive BP cuffs).The Intervention Group will receive the BP cuff and weekly SMS messages asking for the BP reading. The methods section below will outline the details for both groups.

**METHODS:**

***1. Study Instruments:***

The study instrument comprises of a 6-month study visit to measure blood pressure at the conclusion of the study period and the data that is collected by Way to Health.

## *2. Group Modifications:*

Patients in the usual care arm will not be contacted by study staff at the start of the pilot, they will not receive a blood pressure cuff, and they will not receive any text messaging or any component of the program. They will eventually be contacted by study staff to schedule the 6 month BP check. Patients assigned to intervention arm will a text message notifying them of enrollment, be mailed a BP cuff and receive a recruitment phone call if nonresponsive to text. If the patient does not opt out, the research coordinator will mail a blood pressure cuff and proper measurement instructions and start their remote monitoring program in the Way to Health platform. Usual care for hypertension is as needed determined by the clinical expertise of the primary care provider and can include regular follow-up visits (in person or virtual), home blood pressure readings, titration of medications during visits or via telephone, referral to specialty care (e.g., Nephrology or Cardiology), blood tests or imaging studies.  Both arms will continue to receive usual care as directed by PCP. Therefore, there are no risks related to foregoing usual care in the intervention arm or in usual care.

*3. Method for Assigning Subjects to Groups:*

Once participants are eligible to begin the study, they will be randomly assigned to the remote monitoring program or usual care. Allocation will be 1:1. The randomization will be at the patient level and stratified by PCP.

## *4. Administration of Surveys and/or Process:*

## Control Group

## 6 month in person clinic visit

## Intervention Group

## Communication via W2H

## 6 month in person clinic visit

##

## *5. Data Management:*

Data on physicians and patients will be obtained from the Epic instance and Epic’s reporting database Clarity at Penn Medicine. Any information that is obtained will be used for research purposes only. Information on patients will only be disclosed within the study team and to the patient’s primary care physician.. All study staff will be reminded of the confidential nature of the data collected and contained in these databases. To ensure that patient confidentiality is preserved, individual identifiers (such as name and medical record number) are stored in a single password protected system that is accessible to study research, analysis and IT staff only. This system, Way to Health (W2H) is hosted on site at UPenn and is protected by a secure firewall. Once a participant is in this system, they will be given a unique study identification number (ID). The study ID will also be used on all analytical files. Please see link for full W2H privacy and security details: <https://policy.waytohealth.org/>. The blood pressure data sent by participants through W2H will be transmitted via cellular signal to W2H without any subject identifiers. Penn Medicine Academic Computing Services (PMACS) will be the hub for the hardware and database infrastructure that will support the project and where the W2H project web portal is based. PMACS is a joint effort of the University of Pennsylvania's Abramson Cancer Center, the Cardiovascular Institute, the Department of Pathology, and the Leonard Davis Institute. PMACS provides a secure computing environment for a large volume of highly sensitive data, including clinical, genetic, socioeconomic, and financial information. Among the IT projects currently managed by PMACS are: (1) the capture and organization of complex, longitudinal clinical data via web and clinical applications portals from cancer patients enrolled in clinical trials; (2) the integration of genetic array databases and clinical data obtained from patients with cardiovascular disease; (3) computational biology and cytometry database management and analyses; (4) economic and health policy research using Medicare claims from over 40 million Medicare beneficiaries. PMACS requires all users of data or applications on PMACS servers to complete a PMACS-hosted cybersecurity awareness course annually, which stresses federal data security policies under data use agreements with the university. Curriculum includes HIPAA training and covers secure data transfer, passwords, computer security habits and knowledge of what constitutes misuse or inappropriate use of the server.

## STUDY PROCEDURES:

***1. Detailed Description:***

For the intervention arm, after the PCP has had the opportunity to opt out any patients they deem inappropriate, the research team will create encounters in Penn Chart and upload the list to Way To Health including the MRN and CSN. Way To Health will start enrollment for intervention patients and send introduction via text messages. The patient outreach text can be found in patient outreach document.

On a rolling basis as patients’ complete orientation, the research team will mail BP cuffs. For patients who do not complete the text message orientation, the research team will call the patients up to two times. The first phone call will be three business days after the initial outreach and the second call will be seven business days after the initial outreach. If there is no response, the patient will be deemed as “do not contact” and the research team will change their status to inactive in Way To Health.

For patients who get started in the program Way To Health will change their status to “active”. The BP reminder will begin when patients text in “READY”. The notifications will be sent to the research team when the patient starts, and the research team will update Penn Chart with documentation. If the patient does not text back “READY” then the research team will call the intervention patient up to two times, the first being 10 days after mailing and the second being 14 days after mailing

For enrollment documentation when the patient texts “READY” or confirms readiness by phone call a telephone encounter will be created to document patient enrollment. The PCP will then be able to continue to manage their patient as they normally would. Any encounters resulting from interactions via the remote monitoring program will be routed to the PCP inbox. The research team will notify the PCP of any medication changes. If the patient opts out after starting, a telephone encounter will be created, and the research team will be notified and will notify the PCP.

The BP request messaging is in the Patient and Provider materials document that is attached and will be sent weekly on the specified day and time that the patient indicated in the enrollment survey.

The BP Logic categorization is in the below table. Further information on the logic and messaging can be found in the attached Patient and Provider materials document.
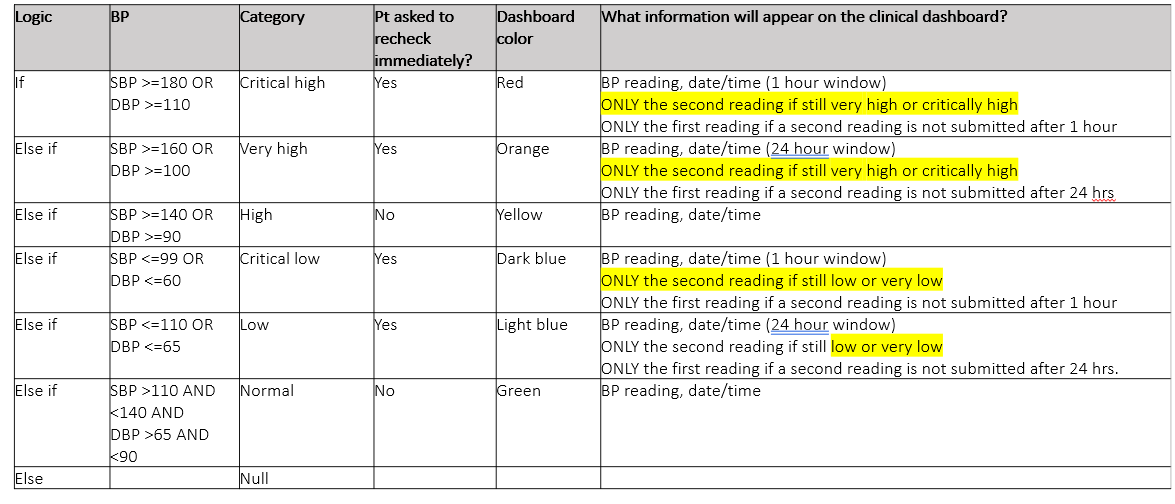


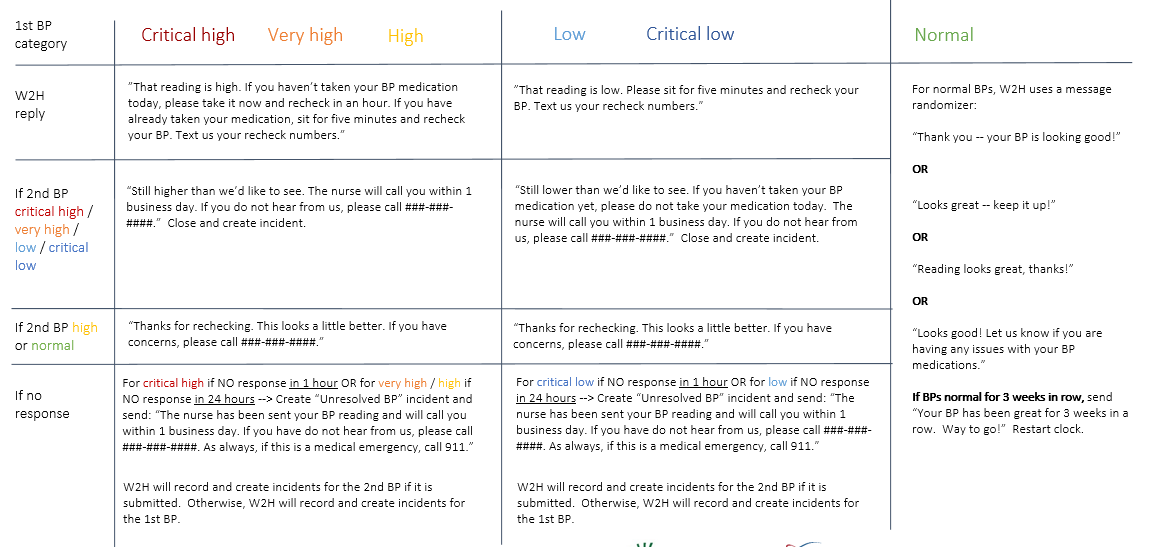


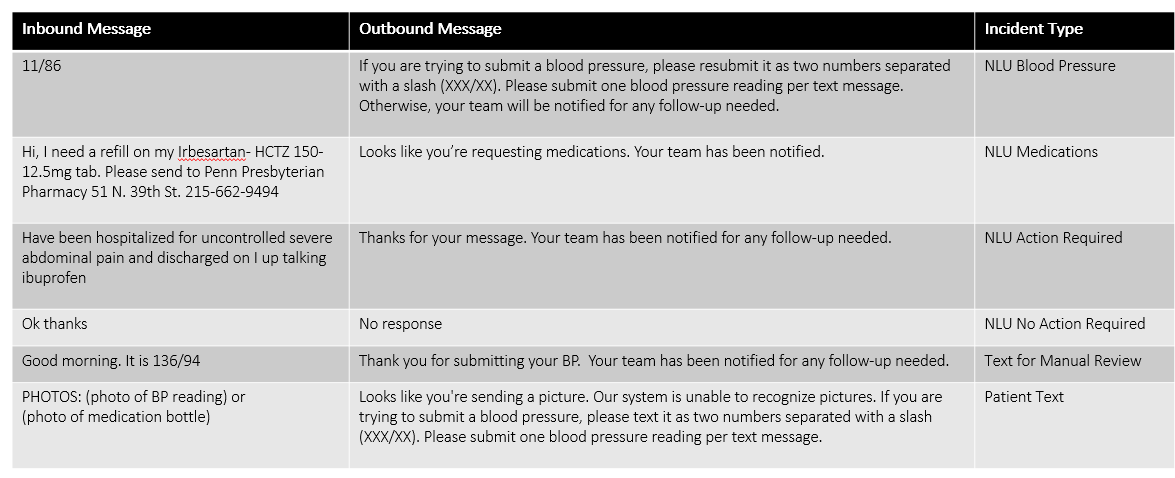


For completion the of the project. Participants will be notified via text. An example of the text can be found in the attached Patient and Provider materials document.

For participant completion the research team will be notified via email. Way To Health will move patient status to finished. The patient will no longer receive texts.

**The research team uses Omni to aggregate the data on the participants. Omni**is a Penn Medicine approved, web-based platform that aggregates information from Way to Health and PennChart (electronic health record) into customizable dashboards that allow users to view information relevant to specific clinical populations. Omni’s use of real-time and retrospective data makes it a scalable platform on which new rules for individual and population-level interventions can be developed, tested, and deployed.

The research team will reach out to the PCPs upon the completion of participants. Example of how PCP will be messaged can be found in attached document of patient messaging.

If participants do not respond to the request for blood pressure three times in a row, then unresponsive logic will set in.

The initial unresponsive text they will receive can be found in the attached document of patient messaging.

The workflow based on unresponsive logic is as follows.


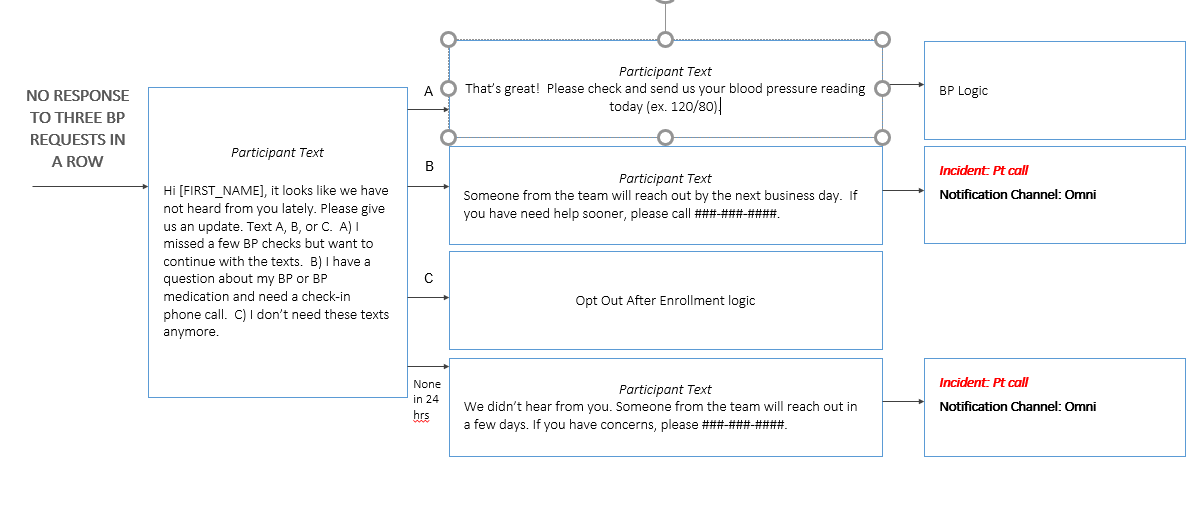


***2. Data Collection:***

The research will include assessing inclusion and exclusion criteria from clinical data in the EPIC/PennChart EMR that has been collected during regular clinical care prior to the study initiation. This data will determine whether the patient is eligible for the study. Data Science will collect this data to identify and generate lists of eligible study participants. Data Science will again collect clinical data from the EPIC/PennChart EMR to assess the primary and secondary outcomes at the end of the 6 month study period for participants in both the usual care and intervention arms. This data will be combined with home blood pressure readings collected by Way to Health to assess the primary and secondary outcomes.

***5. Statistical Analysis:***

**STATISTICAL CONSIDERATIONS**

**Statistical Hypotheses**

- Primary Efficacy Endpoint(s):
  - Hypothesis: The mean decrease in systolic blood pressure from baseline to the 6 month study visit will be 5 mmHg greater in the intervention group relative to the control arm.
- Secondary Efficacy Endpoint(s):
  - Hypothesis 1: Systolic blood pressure will be 5 mmHg lower in the intervention group relative to the control arm at 3 months post randomization, measured pragmatically.
  - Hypotheses 2: Diastolic blood pressure will be 5 mmHg lower in the intervention group relative to the control arm at 3 months (measured pragmatically) and 6 months (study visit) post randomization.
  - Hypothesis 3: The proportion of patients having achieved blood pressure control (as defined by a BP < 140/90) will increase by 10% in the intervention group relative to the control group.

**Sample Size Determination**

**We aim to power our study to detect a mean systolic blood pressure difference of 7 mmHg between study arms at the 6 months study visit. From baseline pilot data we expect a standard deviation around 17. With 80% power and a Type I error rate of 0.05, we estimate that we will need data on about 200 patients to be able to reject the null hypothesis of no difference between groups. Recognizing that there will be a reasonable rate of loss to follow-up, we plan to randomize 300 patients.**

**Statistical Analyses**

***General Approach***

For descriptive statistics, we will present continuous data as means with standard deviation or median with interquartile range, as appropriate. Categorical data will be presented as percentages. For inferential tests, we will use a p-value of 0.05 as the cutoff for statistical significance; tests of hypothesis will be two-tailed.

***Analysis of the Primary Efficacy Endpoint(s)***

The primary analysis will be an intention to treat analysis among those that met inclusion criteria and were randomized to the intervention or control arm. The primary endpoint will be a continuous measure of change in blood pressure from baseline to the 6 month study visit. Crude between group differences will be assessed using t-tests. We will also use a linear regression model. While randomization should create balance between arms, if there is any residual imbalance in covariates deemed to be associated with the outcome, these will be adjusted for to enhance efficiency. These may include: age, sex, race, ethnicity, payor group, Charlson comorbidity index. Patients enrolled in the intervention arm will be included in the analysis even if they opt out of the texting intervention (intention-to-treat). In addition, we will conduct a secondary per-protocol analysis including only those patients randomized to the intervention who completed enrollment, and ultimately received equipment and messages.

***Analysis of the Secondary Endpoint(s)***

Secondary endpoints will include the change in systolic blood pressure at 3 months post randomization; change in diastolic blood pressure measured at 3 months and 6 months; and blood pressure control (<140/90) at 6 months. The statistical approach for continuous measures will be similar to that described above. For the binary outcome of blood pressure control, crude differences will be assessed with a chi-squared test, and we will use a logistic regression model with adjustment for covariates as described above.

***Baseline Descriptive Statistics***

Baseline descriptive statistics will include mean baseline blood pressure and number of anti-hypertensive medications. In addition we will provide information on the covariates described above, including median age and interquartile range; percentages of sex, race, ethnicity, and insurance payor group; and mean Charlson comorbidity index.

***Sub-Group Analyses***

As noted, we will conduct a secondary per-protocol analysis including only those patients randomized to the intervention who completed enrollment, and ultimately received equipment and messages.

## *Data Analysis*

## The baseline is the mean of last two BP. The final BP is the one taken at the six month visit.

**RISK/BENEFIT ASSESSMENT:**

***1. Risks:***

We consider this study minimal risk. The primary risk would be from a breach of confidentiality involving medical record reviews and monitoring of hypertension medication and blood pressure measurement adherence with text messaging which will be maintained on the Way to Health platform. This risk has been mitigated by extensive privacy protection protocols, a highly secure data storage system, and a plan to remove identifiers from the data wherever possible. In addition, all personnel will be held to high standards of upholding confidentiality and safeguarding patient privacy

***2. Benefits:***

The immediate benefits of this study for participants may include an improvement in adherence to behaviors and medications that have been proven to be effective in improving patient outcomes. It is possible that the benefits for many participants will be minimal. However, as mentioned, we believe the risks are also minimal. Knowledge gained from the study will assist in understanding effectiveness of the remote monitoring/chatbot approach.

***3. Subject Privacy:***

All efforts will be made by study staff to ensure subject privacy. The study staff will only interact with the subsample of subjects that are enrolled in the study. Participants will be asked to confirm their identify in the initial text message that is sent. If phone calls are required to help participants set up the blood pressure cuff, troubleshoot bp cuff issues or schedule the bp measurement at end of the intervention period, they will conduct these phone calls in a private area. Whenever the research staff calls the participant, they will confirm their identity before proceeding.

## *4. Subject Confidentiality:*

Confidentiality refers to the subject’s understanding of, and agreement to, the ways identifiable information will be stored and shared.

**How will confidentiality of data be maintained? Check all that apply.**

Paper-based records will be kept in a secure location and only be accessible to personnel involved in the study.

Computer-based files will only be made available to personnel involved in the study through the use of access privileges and passwords.

Prior to access to any study-related information, personnel will be required to sign statements agreeing to protect the security and confidentiality of identifiable information.

Whenever feasible, identifiers will be removed from study-related information.

A Certificate of Confidentiality will be obtained, because the research could place the subject at risk of criminal or civil liability or cause damage to the subject’s financial standing, employability, or liability.

A waiver of documentation of consent is being requested, because the only link between the subject and the study would be the consent document and the primary risk is a breach of confidentiality. (This is not an option for FDA-regulated research.)

Precautions are in place to ensure the data is secure by using passwords and encryption, because the research involves web-based surveys.

Audio and/or video recordings will be transcribed and then destroyed to eliminate audible identification of subjects.

Other (specify):

Data on physicians and patients will be obtained from Penn Medicine system. Any information that is obtained will be used for research purposes only. Information on Primary Care Providers (PCPs) and their patients will only be disclosed within the study team. All study staff will be reminded of the confidential nature of the data collected and contained in these databases. All efforts will be made by study staff to ensure subject privacy. Data will be evaluated in a de-identified manner whenever possible.

The data will be stored and analyzed on the LDI-supported secure Health Services Research Data Center (HSRDC) servers. The HSRDC is comprised of secure high-performance servers within the University of Pennsylvania’s Perelman School of Medicine that have the necessary security protections to permit storage and analysis of data containing Protected Health Information by LDI-affiliated investigators and research staff. The database management on these servers is built with multiple layers of security and follows best practices for securing sensitive data. The main levels of security are fourfold and include machine physical security at the IT facility at which the servers are housed, physical server security in the highly restricted server containment room, electronic server security (firewalls, passwords, and encryption) restricting access to the machine, and directory access controls restricting access to these particular data. The hardware is located in a high-security server center shared by the University of Pennsylvania School of Medicine and University of Pennsylvania Health System. This location eliminates the need for project staff to store or analyze any data on a desktop or laptop computer or use portable media, which generally pose much greater security risks. The system is managed by a team of full-time systems analysts with advanced training in system/network security, and who have established multiple layers of physical and cyber security and system monitoring, in accordance with the standards established by the 2002 Federal Information Security Management Act (FISMA). The HSRDC employs a dedicated queue in an IBM LSF cluster containing three servers for data analysis. The servers are running CentOS Linux 7 and consist of a Dell PowerEdge R820 (4x Intel Xeon E5-4650 v2 2.40 GHz CPU 258GB RAM) and two

Dell PowerEdge R740 (2x Intel Xeon Gold 6138 2.0 GHz CPU 503GB RAM) each.File storage is provided by three dedicated NFS v4 servers on VMware Virtual Machines (8x Intel Xeon E5-4640 @ 2.40 GHz CPU, 8GB RAM) running CentOS Linux 6 (2 hosts) and CentOS Linux 7 (1 host). Job submission and file transfer services are provided by an LSF Submit Host on a CentOS Linux 7 VMware Virtual Machine (2x Intel Xeon E5-4640 @ 2.40 GHz CPU, 4GB RAM). The LSF scheduler runs on two CentOS Linux 7 VMware Virtual Machines (2x Intel Xeon E5-4640 @ 2.40 GHz CPU, 4GB RAM). The dedicated MySQL server runs on a CentOS Linux 6 VMware Virtual Machine (4 x Intel Xeon E5-4640 @ 2.40 GHz CPU, 8GB RAM).

The servers are housed in a dedicated computer machine room containing emergency backup power (e.g., an uninterruptible power system), a non-liquid fire suppression system, and biometric authorization-based room access with a severely restricted physical access list. Software installed on the servers includes the latest versions of SAS, Stata, R, Python, and MySQL, permitting a wide array of analyses including advanced statistics, econometrics, and machine learning techniques.Access to databases and corresponding XYZ data is controlled by the IT facility team to designated users, who will connect to the system using Secure Shell (SSH) and/or Virtual Private Network (VPN) software with mandatory “strong” passwords. Data, query tools, and reports published via web interfaces will be encrypted using a secure web server and SSL certificates that provide a minimum of 1024-bit encryption. The electronic data files for this study will be stored and analyzed solely on this dedicated, layered-security system, which can be accessed only by the PI and designated project staff that are under the direct supervision of the PI.

Since the system is guarded by multiple levels of physical and cyber protection, is monitored by system administrators constantly for unauthorized access, and is accessible only to key personnel, the risk of unlawful penetration is not a significant data safeguard concern.

***5. Protected Health Information***

Throughout the intervention period, an EPIC data analyst from Penn will pull patient records to assess patients who may be eligible for the intervention. A limited data set will be provided to the study team. This patient care data will be examined to determine if the patient is eligible for the intervention. EPIC patient care data will be used to assess patient adherence. Patient PHI will be collected to assess whether the intervention led to increased adherence.

- Name
- Street address, city, county, precinct, zip code, and equivalent geocodes
- All elements of dates (except year) for dates directly related to an individual and all ages over 89
- Telephone
- Medical record numbers

The following entities, besides the members of the research team, may receive protected health information (PHI) for this research study: Greenphire ClinCard, the company which processes study related payments. Patient addresses and account balances will be stored on their secure computers. Twilio, Inc., the cloud communications platform integrated with the Way to Health platform and is the messaging service which processes some study-related messages. Twilio will store de-identified patients' phone numbers and message content on their secure computers. Qualtrics, Inc., the company which processes most study-related surveys. Qualtrics will house de-identified answers to these surveys on their secure servers. The Office of Human Research Protections at the University of Pennsylvania -Federal and state agencies (for example, the Department of Health and Human Services, the National Institutes of Health, and/or the Office for Human Research Protections), or other domestic or foreign government bodies if required by law and/or necessary for oversight purposes.

***6. Compensation:***

Participants will be compensated $75 for coming in at 6 months for a clinical blood pressure measurement. At the of the intervention, participants in the intervention arm will be able to keep their blood pressure cuff.

***7. Data and Safety Monitoring:***

The Principal Investigator will be responsible for monitoring the study. All participants will be given anticipatory guidance on when to seek medical attention. In addition, participants will be asked to report to the study team any injuries or medical care that they feel resulted from participation in the study. They can either call the study team or send an email. There will be a research landing page whose web link will be included in the initial welcome text from WTH. The webpage messaging is included in an attachment. The research coordinator will call the participant to collect information regarding the issue and then the PI will review and determine whether it is ok to proceed, further investigation is needed, or the participant should stop the study. For this study there will be no stopping rules or endpoints and thus no planned interim analyses.

***8. Investigator’s Risk/Benefit Assessment:***

This study is designed to test effectiveness of a remote monitoring program that incorporates many components of prior interventions that have previously demonstrated promise in feasibility, clinical workflow, and potentially relevant improvement in BP. We believe the combination of these approaches in this intervention will provide the research and public health communities with important information that can lead to broad generalizability in treating people at risk for the above-mentioned chronic diseases and death nationally, as these types of programs could be set up by healthcare systems to be broadly utilized. However, successful recruitment and engagement of patients is critical to the success of the program itself, particularly in a remote management context. With minimal risks, the potential public health impact of a successful recruitment to a program to improve BP control is great and could reduce the number of deaths from health-related outcomes in the United States each year.

***INFORMED CONSENT:***

***1. Consent Process:***

This pilot will be conducted under a waiver of the requirement for informed consent based on the following criteria set for forth by the Federal Policy for the Protection of Human Subjects (the “Common Rule”):

1. The research involves no more than minimal risk to subjects.
2. The waiver will not adversely affect the rights and welfare of the subjects.
3. The research cannot be practicably conducted without a waiver of the requirement for informed consent.

**The research involves no more than minimal risk to subjects:** The risks to subjects of participating in this study is no more than minimal as both arms will be receiving usual care as determined by PCP. The intervention arm will exceed this standard of care by including a remote monitoring program. This service is provided free of charge to patients, with the exception of any standard SMS charges from their carrier, about which the patient will be notified upon enrollment. Patients in either arm will be free to contact their practice, or seek care elsewhere, in the usual manner and in any way they see fit.

**The waiver will not adversely affect the rights and welfare of the subjects:** The intervention arm of this study (those randomized to receive automated text messages) does not impact patients’ ability to receive usual care, as determined by PCP. As noted, patients will be able to easily opt out of text messages at any time, and will be provided with clear instructions on doing so. A link will be provided in the introductory text message to a section of the Way To Health website, describing the program, and indicating that patients’ data may be anonymized and used to study the impact of the program. This page will provide the contact information for a research coordinator if the patient wishes to not have their data analyzed for research purposes. The messaging on the landing page can be seen in the attached Patient and Provider materials document.

**The research cannot be practicably conducted without a waiver of the requirement for informed consent:** The pilot seeks to evaluate the effectiveness of adding automated SMS program to the existing standard of care. Requiring individual informed consent will introduce significant selection biases. Whereas in drug trials the primary questions are related to physiologic responses, in behavioral trials such as this, individuals who consent are likely to have significantly different behavioral characteristics from those who don’t, and therefore would not be representative of the overall population, biasing the outcomes of the study.

**RESOURCES NECESSARY FOR HUMAN RESEARCH PROTECTION:**

Team is adequately informed of the protocol and adequately qualified to conduct research via training required for medical doctors/students and research coordinators. All are up to date with HIPAA and CITI training.

Detailed Standard Operating Procedure documents for the project will be accessible to all members of the research team, which will keep research staff informed about the protocol and their related duties. There are adequate facilities to conduct the research.

## References

1. Dai H, Bragazzi NL, Younis A, et al. Worldwide trends in prevalence, mortality, and disability-adjusted life years for hypertensive heart disease from 1990 to 2017. *Hypertension.* 2021;77(4):1223-1233.
2. Fryar CD, Ostchega Y, Hales CM, Zhang G, Kruszon-Moran D. Hypertension prevalence and control among adults: United States, 2015-2016. 2017.
3. Muntner P, Hardy ST, Fine LJ, et al. Trends in blood pressure control among US adults with hypertension, 1999-2000 to 2017-2018. *JAMA.* 2020;324(12):1190-1200.
4. Eberly LA, Sanghavi M, Julien HM, Burger L, Chokshi N, Lewey J. Evaluation of Online Patient Portal vs Text-Based Blood Pressure Monitoring Among Black Patients With Medicaid and Medicare Insurance Who Have Hypertension and Cardiovascular Disease. *JAMA network open.* 2022;5(2):e2144255-e2144255.
5. Hirshberg A, Downes K, Srinivas S. Comparing standard office-based follow-up with text-based remote monitoring in the management of postpartum hypertension: a randomised clinical trial. *BMJ quality & safety.* 2018;27(11):871-877.
